# An In Vitro Study of the Effects of Methotrexate Loaded Biocomposite Beads on MG63 Osteoblast Cells

**DOI:** 10.1038/s41598-025-85702-y

**Published:** 2025-01-17

**Authors:** A. E. Malash, A. A. Al-esnawy, Khairy T. Ereiba, Ahmed M. Bakr, A. S. Abdraboh

**Affiliations:** 1 Physics Department, Faculty of Science, Al-Azhar University, Nasr City, Cairo, 11884 Egypt; 2 Spectroscopy Department, Physics Research Division, National Research Centre, 33 El Bohouth St., Dokki, Giza, 12622 Egypt

**Keywords:** Drug carrier, Cell culture, Cell line, Bioactivity, Bioglass, Beads, Drug delivery, Biophysics, Cancer, Materials science

## Abstract

This study aims to synthesize a new localized drug delivery system of bioglass, polyvinyl alcohol (PVA), cellulose (CNC), and sodium alginate (SA) beads as a carrier for methotrexate (MTX) drugs for the treatment of osteosarcoma. Methotrexate /Bioglass-loaded Polyvinyl/Cellulose/Sodium alginate biocomposite beads were prepared via the dropwise method with different concentrations of (65%SiO_2_-30%CaO- 5%P_2_O_5_) bioglass. Samples were named B0, S0, S1, S2, and S3, respectively. Calcium chloride (CaCl_2_) was used as a cross-linking agent. The obtained biocomposite beads were investigated by different techniques FTIR, XRD, SEM, etc. The bioactivity of MTX/BG-loaded PVA-CNC-SA biocomposite beads was tested by immersion in simulated body fluid (SBF). The profile release of methotrexate was investigated with UV–vis spectroscopy for 30 days. A cytotoxicity study of the methotrexate was performed by a human osteosarcoma (MG-63) cell line. Results indicated that the formation of a hydroxyapatite layer on the bead’s surface confirmed its biological activity. Bioactivity was directly proportional to the BG content. All samples of B1, S0, S1, S2, and S3 exhibited significant maximum release up to 6 days and were controlled gradually. Cytotoxicity results of biocomposite beads showed that high cell death was detected on the MG-63 cells, with (IC-50 ± SD) of S3 (116.16 ± 1.57) compared with B1 (306.99 ± 2.72) and S1 (204.74 ± 4.55) due to the high release of MTX, which was confirmed by the results of the drug release profile. Results prove that the prepared biocomposite beads can be used as bioactive, drug delivery systems, and anticancer materials.

## Introduction

Primary bone tumors, particularly osteosarcoma, are among the most dangerous due to their tendency to metastasis to other parts of the body, such as the lungs, breast, and prostate^1,2^*.* Even after thorough clinical treatment, the five-year overall survival rate for osteosarcoma patients remains below 60%^3^. Initially, the standard treatment for osteosarcoma is surgery, but it significantly impacts patients’ physical and mental health. Osteosarcoma primarily affects children, adolescents, and adults. Chemotherapy is currently a critical and viable clinical treatment strategy to slow the progression of osteosarcoma, with methotrexate playing a central role since the 1970s^4,5^. However, MTX drugs have limited solubility in water, pharmacokinetics, and low bioavailability limiting their clinical effectiveness. High-dose MTX pulse therapy often leads to drug resistance and serious side effects, including immune suppression, myelosuppression, hepatotoxicity, and cardiotoxicity^6,7^. Efficient drug delivery is crucial in cancer therapy. Recently, targeted therapy has gained significant attention, aiming to deliver drugs to specific organs through a guided mechanism, enhancing treatment efficacy and reducing side effects. This approach, often called the “magic bullet,” focuses on drug delivery systems that target tumor tissue, potentially offering a more effective cure for tumors^8^.

Methotrexate is a well-established chemotherapeutic and immunosuppressive agent that has been extensively utilized in the treatment of various malignancies, autoimmune disorders, and inflammatory conditions. It functions as an antimetabolite by inhibiting dihydrofolate reductase, a critical enzyme in folate metabolism, thereby disrupting DNA synthesis and cell replication. Despite its clinical efficacy, the therapeutic application of MTX is often constrained by systemic toxicity and suboptimal biodistribution. Consequently, innovative drug delivery systems, such as polyelectrolyte complexes (POECs), have been explored to address these limitations. POECs offer the potential to enhance MTX delivery through targeted release mechanisms, minimizing off-target effects and improving therapeutic outcomes. Studies demonstrate the effectiveness of MTX-loaded nanoparticles, fabricated using POECs, in controlled and sustained drug release, thereby underscoring its significance in precision medicine and targeted cancer therapies^9^.

Bone cancer treatments involve tumor resection, followed by reconstructing the defect with biomaterials. Bioactive glass is a highly effective material for hard tissue repair and tissue engineering due to its excellent biocompatibility, biological mineralization properties, and appropriate degradation rate^10,11^. When combined with drugs, bioactive glasses can target cancer cells, and osteoclasts, or be used in new therapies such as gene delivery and bioinorganic. The abundant silicon hydroxyl groups of BG allow for easy functional modification, enabling the coupling of targeting molecules and anticancer drugs. This makes BG a promising novel drug carrier for targeted drug delivery systems, thanks to its bioactivity, biocompatibility, and biodegradability^12,13^*.*

In recent years, many notable polymers, such as chitosan^14,15^, cellulose^16^, and polyaniline^17^, have been used for creating functional nano-biocomposite hydrogels. Polyvinyl alcohol is an affordable and biocompatible water-soluble semi-crystalline polymer rich in hydroxyl groups capable of forming hydrogen bonds^18^*.* Its excellent mechanical strength and biocompatibility make it extensively used in the drug delivery process^19^*.* However, PVA gel beads have poor water resistance, prompting researchers to enhance mechanical strength and stability by interpenetrating them with other polymers. Sodium alginate, a non-toxic and natural compound, is often combined with PVA^20^*.* PVA/SA hydrogels have been widely studied for their combined benefits, but their applications are limited by their insufficient stability and mechanical strength. Increasing interest in biocomposite materials has led to various strategies to improve gel strength, such as adding nano-fillers to SA/PVA hydrogels^21^. Cellulose nanocrystals, derived from abundant renewable natural resources, have large surface areas and strong mechanical characteristics^22–24^. Incorporating CNCs into SA membranes enhances their strength, adsorption sites^25^*.* Thus, improving stability and strength^26^. Integrating bioactive glass with PVA-CNC-SA polymers to develop bio-composites shows significant potential for advancing osteosarcoma treatment. These hybrid materials can be engineered to provide a supportive matrix for bone tissue regeneration while serving as a repository for localized and sustained delivery of methotrexate^27^.

Beads, particularly hydrogel beads, are a promising solution in drug delivery systems due to their distinctive properties. These beads can be synthesized from biocompatible polymers like chitosan, which offers pH sensitivity, high swelling capacity, and excellent drug storage capabilities. Their three-dimensional porous structure allows for efficient drug loading and controlled release, tailored to specific environments, such as acidic conditions often found in cancerous tissues. Additionally, integrating nanoparticles like magnetic graphene quantum dots enhances the beads’ stability and enables targeted delivery using external magnetic fields. These attributes make hydrogel beads an advanced tool for improving therapeutic outcomes while minimizing side effects^28,29^.

In this study, we prepared biocomposite beads as a methotrexate-carrying and bioactive material for use in tissue engineering. The MTX/BG-loaded PVA-CNC-SA biocomposite beads were prepared by a dropwise method with different concentrations of bioglass (BG). The bioactivity of these biocomposite beads was tested by immersion in (SBF). The biocomposite beads were analyzed before and after immersion in the SBF solution using various techniques. In vitro studies of samples were conducted on MG-63 osteosarcoma cells.

## Materials and methods

TEOS (Tetraethyl orthosilicate: C_8_H_20_O_4_Si, Mw = 208.33 g/mol), calcium nitrate tetrahydrate (Ca (NO_3_)_2_⋅4H_2_O, Mw = 236.149 g/mol), TEP (Triethyl phosphate: C_6_H_15_O_4_P, Mw = 182.15 g/mol), and 2M nitric acid (HNO_3_) were procured from **Merck Inc (Darmstadt, Germany)**. Acetic acid (96%), Sodium alginate (SA: NaAlg, Polyvinyl alcohol (PVA: (C_2_H_4_O) n), Cellulose nanocrystal (CNC), Calcium Chloride (CaCl_2_) were obtained from **Acros Organic Ltd (New Jersey, USA)**, and Methotrexate (MTX: C_20_H_22_N_8_O_5_, Mw = 454.45g/mol) was bought from **SPH Sine Pharmaceutical Laboratories Co. Ltd**. (H3102067804, Shanghai, China). All other chemicals required for preparing simulated body fluid (SBF) and phosphate-buffered saline (PBS) were purchased from **Sigma-Aldrich (St. Louis, MO, USA)**.

### Synthesis of bioactive glass (65S-BG)

**Fig. 1:**
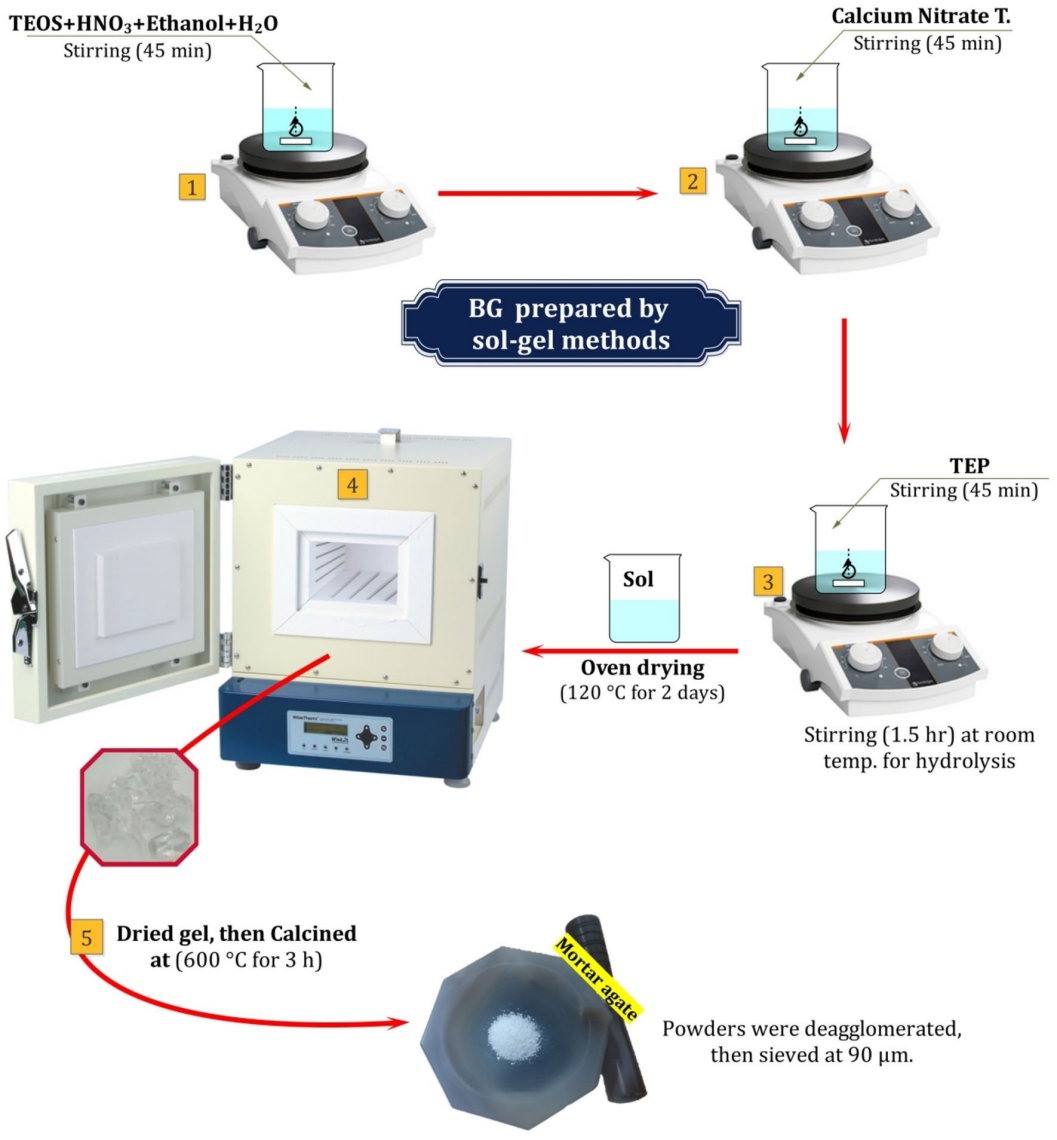
Flow chart of the manufacturing processes for bioactive glass (65S-BG). Created in EdrawMax. Alesnawy, A. (2024), Version: 14.1.0, https://www.edraw.ai/app/max/preview/0atefELMztbKXnlcJUXwpAKgpferMDhn?ivt=c6013b9050acd863d2aa82336c5123500701f9eba1e6c2d0d8c56f36601668e2c7f6b8d8eb83240111862e0bc6cc5cfe.

The bioactive glass was prepared by a sol–gel technique with an average ratio of (65%SiO_2_-30%CaO- 5%P_2_O_5_) powder was synthesized according to *(Al-esnawy *et al*., 2021)*^30^*.* Briefly, 24.4341 ml of TEOS was combined with 4.2222 ml of 2M nitric acid (HNO_3_), and the mixture was allowed to react for 30 min to facilitate the acid hydrolysis of TEOS. Subsequently, the following reagents were added sequentially, with each allowed to react for 45 min: 1.2091 ml of TEP, and 12.8912 ml of calcium nitrate tetrahydrate (Ca (NO_3_)_2_⋅4H_2_O). Then, the mixture kept stirring for 1.5 h until hydrolysis. The solution was heated at 120 °C for 3 days to evaporate all the water. After that, the powder was heated for 3 h at 600 °C to eliminate harmful nitrate ions and integrate calcium into the silicate structure^31^*.* 65S-BG powder was dispersed using an agate mortar. Then, 65S-BG powders were sieved at 90 µm, as shown in the following flow chart, which illustrates the different steps Fig. 1.

### Synthesis of MTX/BG loaded PVA-CNC-SA biocomposite beads

**Fig. 2:**
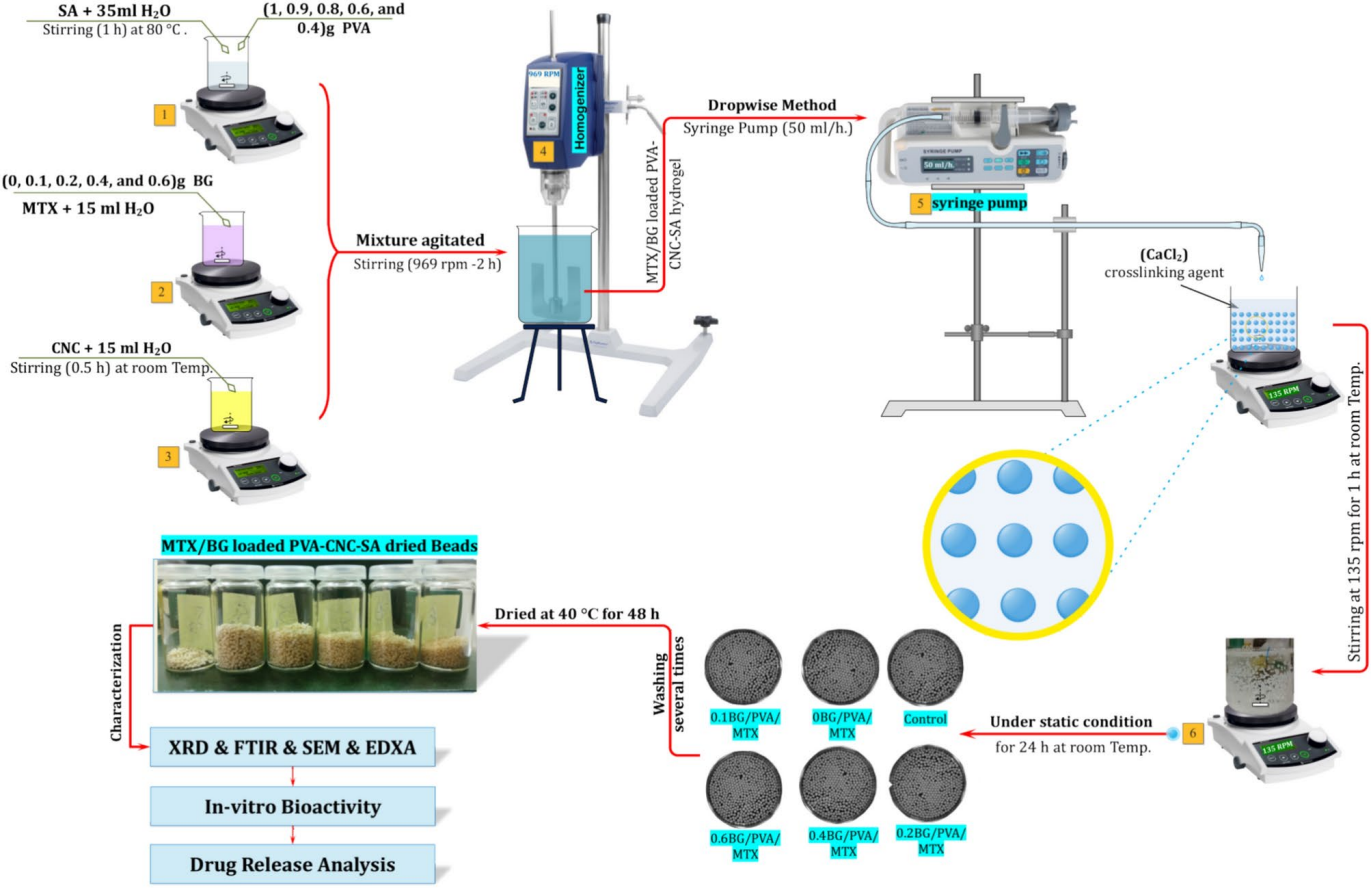
Flow chart of the synthesis of MTX/BG loaded PVA-CNC-SA composites beads. Created in EdrawMax. Alesnawy, A. (2024), Version: 14.1.0, https://www.edraw.ai/app/max/preview/0aGP8464AmEUUWurynpY4kvRS4G2zMm6?ivt=993b7fdc2b4a24707dc5a0a5676145dce03a0ebadd7e4383ef385654d4bac9d2e4c840291cb789df61648ad21a289c8c.

The MTX/BG loaded PVA-CNC-SA biocomposite beads were synthesized following the methods outlined in Fig. 2.

***Solution A:*** 1 g of Sodium alginate and different concentrations of PVA were stirred in 35 ml of deionized water at room temperature.

***Solution B:*** 0.6 g of MTX and different ratios of BG (0/0.1/0.2/0.4/0.6) g were stirred in 15 ml of deionized water at room temperature for 1 h.

***Solution C:*** 1 g of CNC was stirred in 15 ml of deionized water for 0.5 h at room temperature, and the mixture of solutions A, B, and C was homogenized using a homogenizer with a rotating speed of 969 rpm [High-Torque Digital Overhead Stirrer, “HT-50DX” homogenizer (Germany)] for 2 h until the solvent had completely homogenized.

Calcium chloride (CaCl_2_) was used as a cross-linking agent (13.75 g of CaCl_2_ in 250 ml of deionized water)^32^. By the drop-wise method, the (MTX/BG-loaded PVA-CNC-SA) was dropped using a syringe unit pump (50 ml/h) in 250 ml of prepared CaCl_2_ solution to produce biocomposite beads and stirred for 2 h at room temperature as listed in Table 1.

**Table 1:** Chemical composition of MTX/BG loaded PVA-CNC-SA Beads in (wt %).

Sample Code		PVA (g)	CNC (g)	MTX (g)	BG (g)	SA (g)
Control	B0	1	1	0	0	1
0BG/PVA/MTX	B1	1	1	0.6	0	1
0.1BG/PVA/MTX	S0	0.9	1	0.6	0.1	1
0.2BG/PVA/MTX	S1	0.8	1	0.6	0.2	1
0.4BG/PVA/MTX	S2	0.6	1	0.6	0.4	1
0.6BG/PVA/MTX	S3	0.4	1	0.6	0.6	1

The prepared beads were kept under stirring at static conditions for 24 h at room temperature, then removed from the CaCl_2_ solution and washed several times with deionized water. The beads were left to dry in a petri dish at 40 °C and then collected^30^.

### In vitro bioactivity study

The bioactivity of MTX/BG-loaded PVA-CNC-SA biocomposite beads was conducted by immersing them in (SBF) according to the established protocol outlined by *(Kokubo & Takadama, 2006)*^33^. Each sample, weighing 1 g, was submerged in 40 mL of SBF solution in sealed containers for a duration of 30 days at 37 °C. After removal from the SBF solution, the samples underwent rinsing with distilled water, followed by air-drying.

### Characterization

The powder samples of MTX/BG-loaded PVA-CNC-SA biocomposite beads before and after immersion in the SBF solution were investigated using several techniques. X-ray powder diffractometer (XRD); model (BRUKER Germany, D8 ADVANCE) with a copper target (Cu kα = 1.54060 A°) and nickel filter, ranging 2θ from 0° to 60° using a step size of 0.014° with 1 s per step. Fourier-transformed infrared spectroscopy (FTIR) (Nicolet 6700, Thermo-Scientific, USA), Field Emission Gun Scanning Electron Microscopy (FEG-SEM) (XL30, Philips). Energy dispersive X-ray analysis coupled to the SEM instrument (EDXA; 30 mm^2^ Si (Li) R-RSUTW detector) at 15 kV acceleration voltages, and MTX release using UV–VIS spectroscopy (JASCO v-630).

### Cell culture

The in vitro experiments were conducted using MG-63 cells (homo sapiens, human bone, morphology fibroblast, osteosarcoma disease) obtained from Science Way for Scientific Research and Consultations, Cairo, Egypt. MTT assay: The cytotoxicity of B1, S1, and S3 on [1 × 10^5^ of MG-63 cells / ml (100 µl/well), incubated at 37 °C for 24] hours were assessed by the MTT [3-(4,5-dimethylthiazol-2-yl)-2,5-diphenyltetrazolium bromide] assay^34,35^. Cell viability results of composite beads were plotted and analyzed by OriginPro (OriginPro 2024b, OriginLab Corporation, Northampton, Massachusetts, USA) and Statistical software IBM—SPSS, version 27.0

## Results and discussion

### XRD of the biocomposite Beads Before and After SBF

**Fig. 3:**
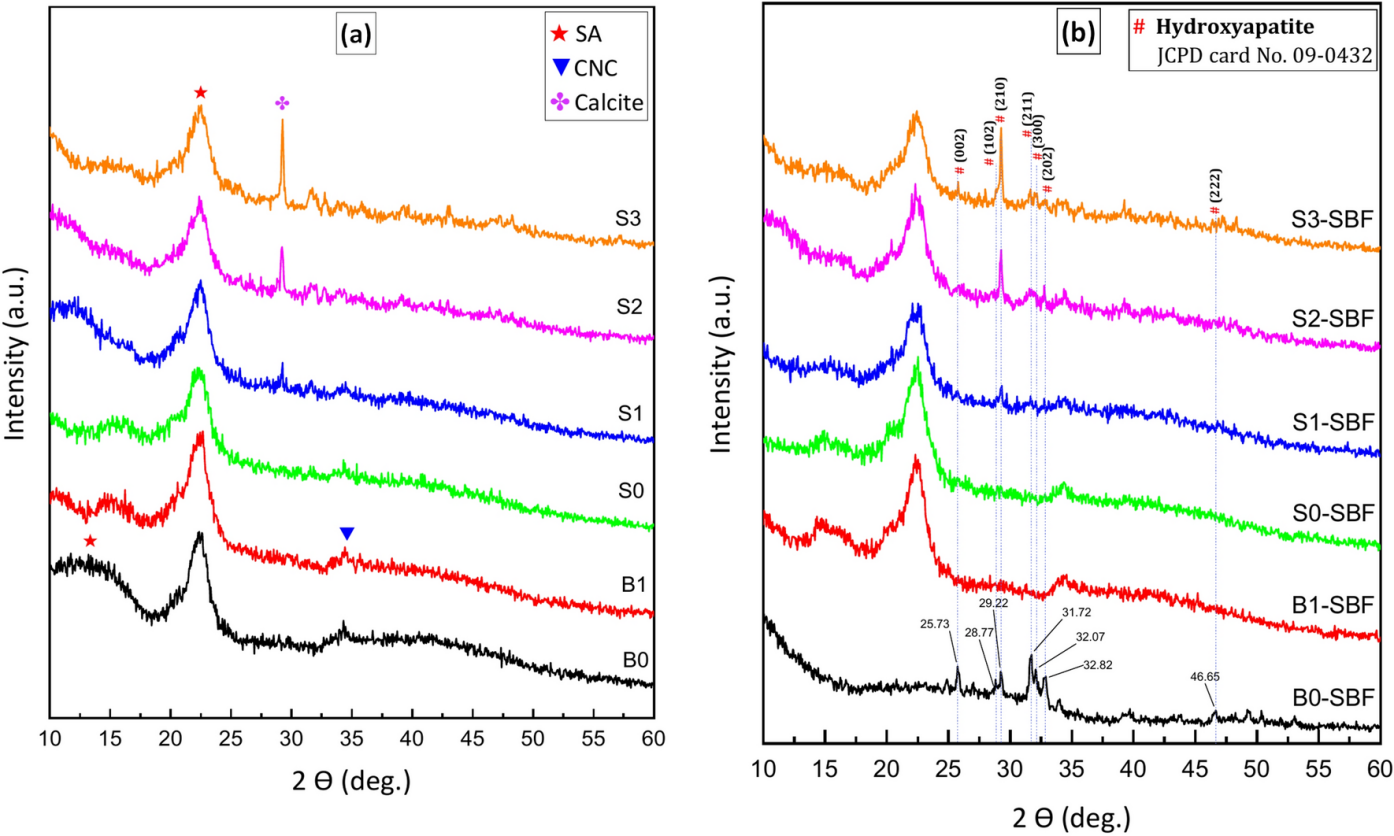
(**XRD**) patterns of MTX, BG, PVA, CNC, and SA composite beads (**a**) before, and (**b**) Following 30 days of immersion in SBF.

Figure 3(a) reports the XRD patterns of all MTX/BG-loaded PVA-CNC-SA beads before immersion in SBF. Three diffraction broadenings (amorphous holes) were observed at 2θ values of (13º and 22.5º) indicating sodium alginate. The broad holes observed in the XRD results, and the absence of sharp peaks indicate the semi-crystalline nature of nanocrystalline sodium alginate and the amorphous state of the prepared beads. The intensity of the observed halos of SA was decreased gradually by the addition of BG content from B0 to S3. One sharp peak at (29º) was observed in S1, S2, and S3 due to the interaction of calcium in BG and carbon in polymers. One weak peak was revealed at 34.8º in B0, B1, and S0, indicating nanocrystalline cellulose^36–38^.

Figure 3(b) reports the XRD patterns of all MTX/BG-loaded PVA-CNC-SA biocomposite beads after immersion in SBF for 30 days. Three strong peaks at 2θ values of (HA found at 2θ of 31.72°, 32.07°, and ~ 32.82°) were observed in (B0) indicated to (HA) according to JCPD card No. 09-0432. Also, the appetite peak intensity was increased gradually from S1 to S3^39^.

### FTIR of the biocomposite beads

FTIR spectroscopy was employed to evaluate the interactions among MTX, BG, PVA, CNC, and SA biocomposite beads, as well as the formation of a hydroxyapatite (HA) layer on the MTX/BG loaded PVA-CNC-SA biocomposite beads’ surface following immersion in SBF.

**Fig. 4:**
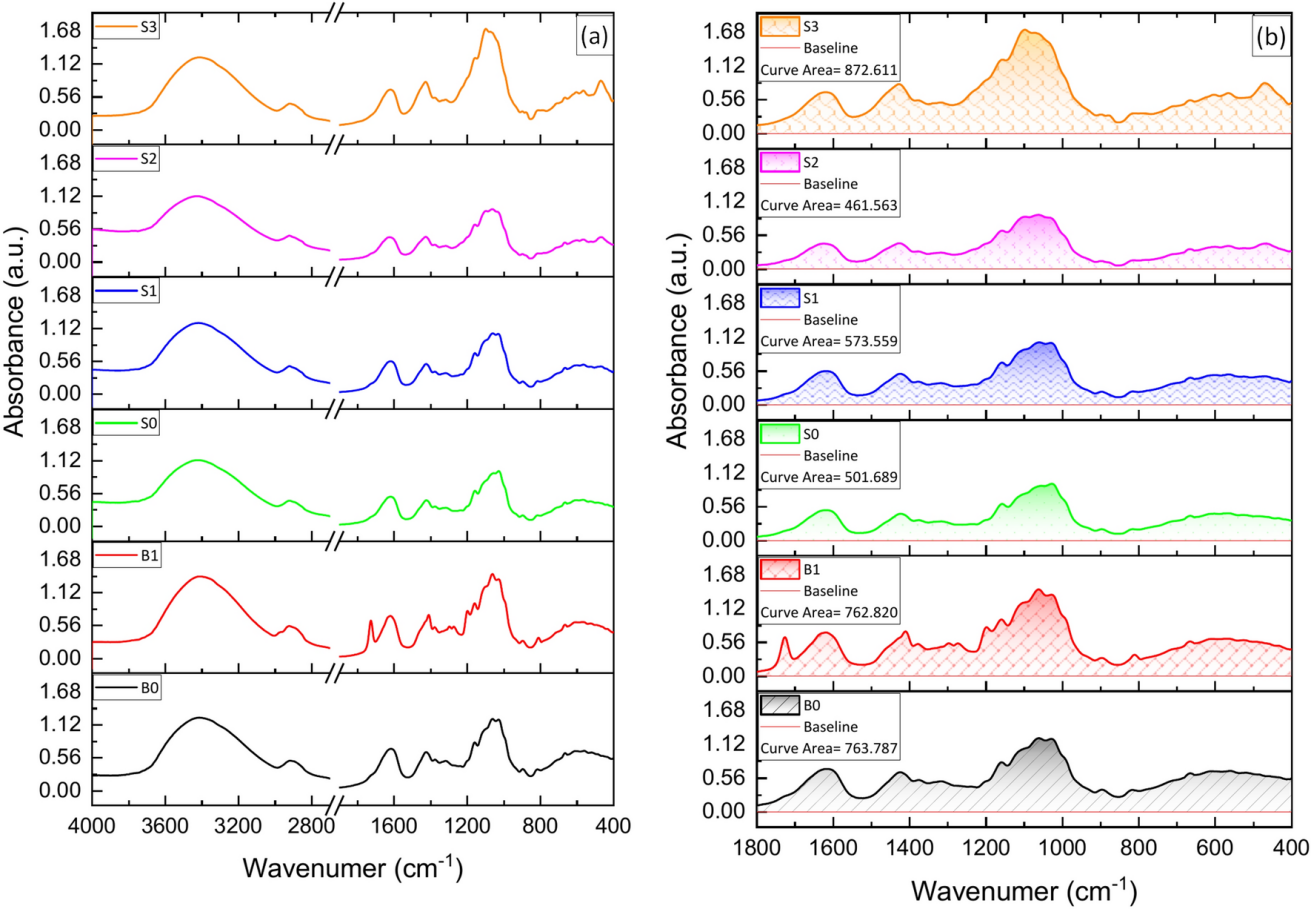
(**a**): FTIR patterns of MTX, BG, PVA, CNC, and SA biocomposite beads before immersion in SBF, (**b**): Area under the curve in the range 400–1800 cm^−1^.

FTIR spectra (Fig. 4) revealed common peaks among SA, PVA, and CNC due to their similar chemical structures. Notably, a prominent peak ranging from 1026 to 1063 cm-1 indicated the overlapping of C-O tension and C–C skeleton vibration peaks^32^. The peak at 1072 cm^-1^ was ascribed to the tensile vibration of C-O^40^. Two distinct bands at 1616 and 1427 cm^-1^ were assigned to the asymmetric and symmetrical stretching of -COO- in SA.

Additionally, the band at 1428 cm^−1^ corresponded to the symmetric bending mode of CH_2_. The C–O–C stretching vibration at the β-(1,4)-glycosidic linkages emerged at 1159–1160 and 896 cm^−1^, while the C–C and C–O skeletal vibrations were observed at 1317 and 1335 cm^−1^ ^41,42^*.* A peak at 1377 cm^–1^ indicated CH bending^43^. Furthermore, two additional peaks at 2960 and 2850 cm^-1^ were associated with the vibration of CH_3_ and CH_2_^32^ .

Characteristic bands of Bioglass were detected at 1027 and 480 cm^-1^, related to Si–O–Si and Si–O stretching modes, respectively^44–46^. Moreover, bands at 1529.24 cm^−1^ and 1616.36 cm^−1^ were attributed to the aromatic C = C in the MTX structure. The infrared spectrum of MTX/BG loaded PVA-CNC-SA biocomposite beads exhibited an absorption band at 3200–3600 cm^-1^ corresponding to the stretching vibration of –NH_2_, C–O, and –OH groups^47^*.*

It is noted that the main characteristic bands of BG, which appeared at 1027 and 480 cm^-1^, increased gradually from S0 to S1 with the addition of BG. Also, common characterization bands at 1030–1072 cm^-1^, 1432–1631 cm^-1^, and 3200–3600 cm^-1^ related to PVA, CNC, and SA decreased gradually as the BG content increased, as confirmed by the area under the curve described in Fig. 4b.

**Fig. 5:**
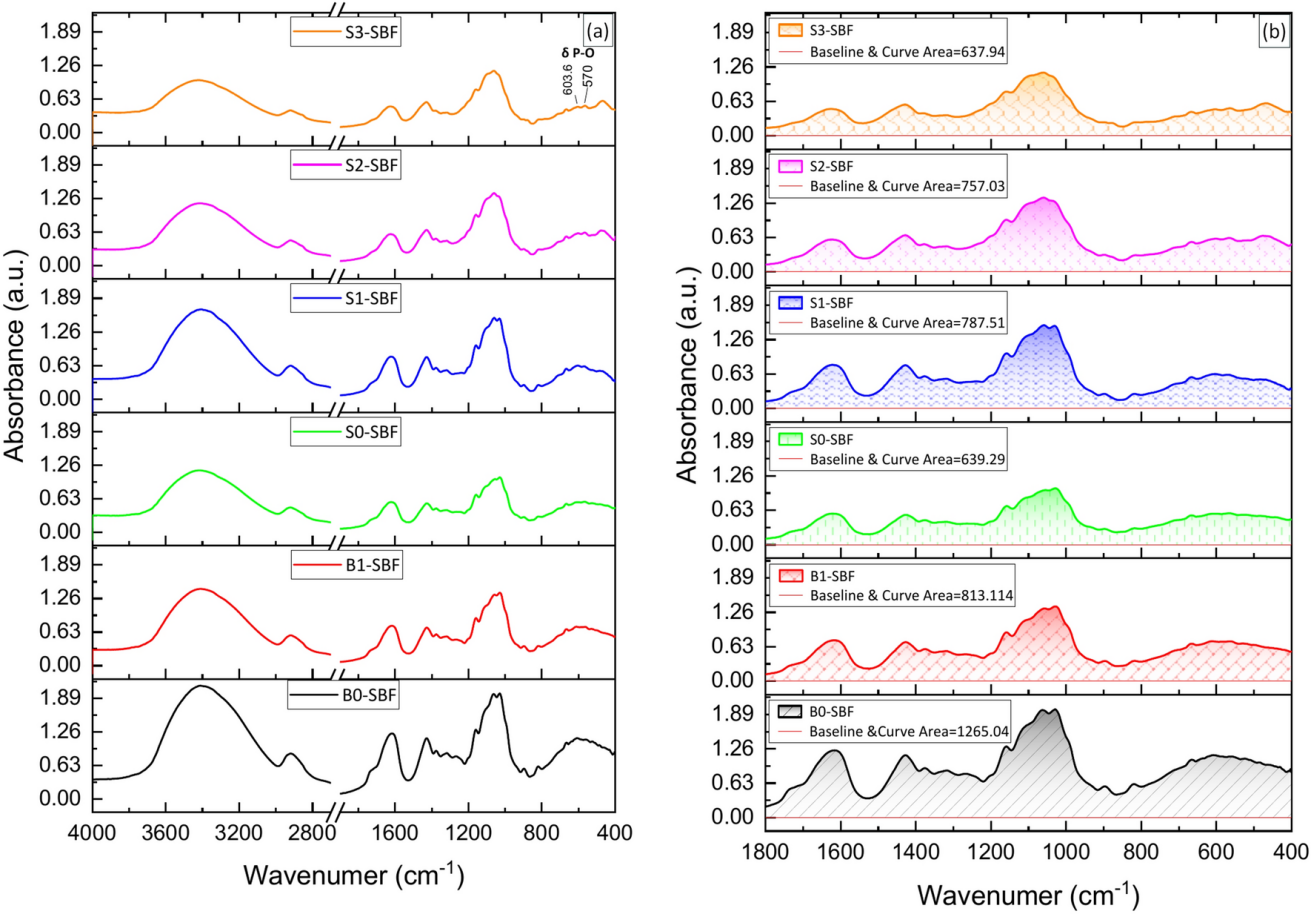
(**a**): FTIR patterns of MTX, BG, PVA, CNC, and SA biocomposite beads After immersion in SBF for 30 days, (**b**): Area under the curve in the range 400–1800 cm^−1^.

After immersion in SBF Fig. 5, The FTIR spectra of MTX/BG loaded PVA-CNC-SA biocomposite beads showed that the silicate network decreased due to the degradation of silica. Two main characteristic bands of (HA) appeared at 570 and 603 cm^-1^. The main band appeared clearly in (S3), which indicates the bioactivity of this sample and the deposition of an HA layer on its surface. The deposition of the calcium phosphate layer on B0 and B1 may be due to the interaction between beads and the crosslinker solution CaCl_2_. The variation in intensities of the silica network and polymer bands is described in Fig. 5b. The variation in the area under the curves in Figs. 4b and 5b is due to the degradation of the biological polymers (PVA, CNC, and SA) and the emergence of new bands corresponding to the deposition of HA.

### SEM & EDXA

**Fig. 6:**
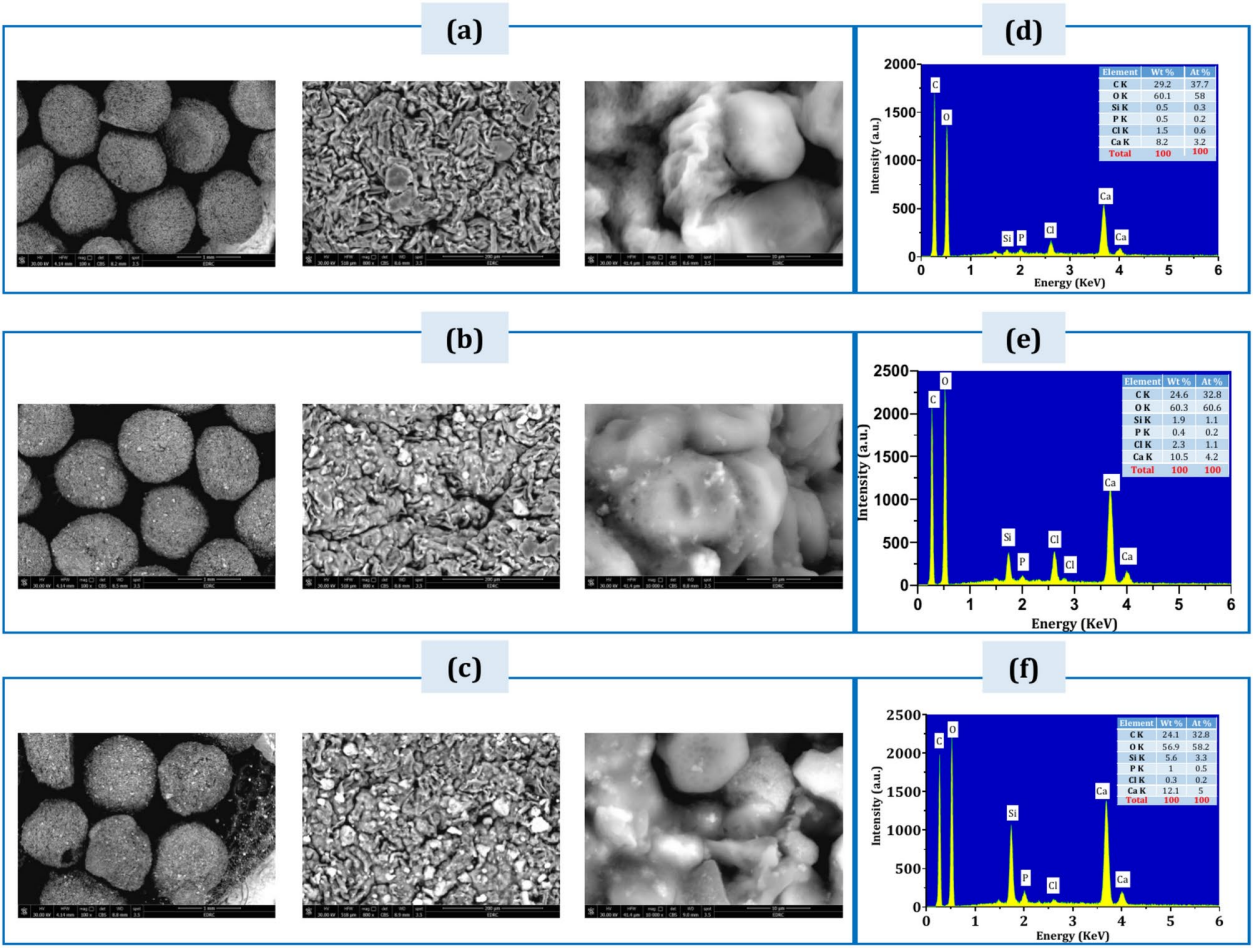
Scanning Electron Microscopy (SEM) images of, (**a**) B1, (**b**) S1, (**c**) S2, and EDXA profiles of (**d**) B1, (**e**) S1, (**f**) S2. Before immersion in SBF.

Scanning electron microscopy (SEM) was employed to analyze the surface structure of MTX/BG-loaded PVA-CNC-SA biocomposite beads prior to immersion in (SBF). Figure 6 shows the SEM of (a) B1, (b) S1, and (C) S2 before the in vitro test (SBF). It can be found that the beads are spherical and uniform in size. The average diameter of beads is about 700 µm. Also, micropores can be found on the surface of the beads, and porosity decreases while BG contents increase.

Figure 6 shows the EDXA profiles of (d) B1, (e) S1, and (f) S2 before the in vitro test (SBF). It is found that the contents of Si, Ca, and P increased gradually from B1 to S2 due to the increased BG ratio. The presence of calcium and chloride in (B1) is due to the cross-linker solution (CaCl_2_). Also, the carbon content indicates the carbon chain of the polymer (PVA, SA, CNC), was decreased due to the polymer ratio decreasing gradually from B1 to S2.

**Fig. 7:**
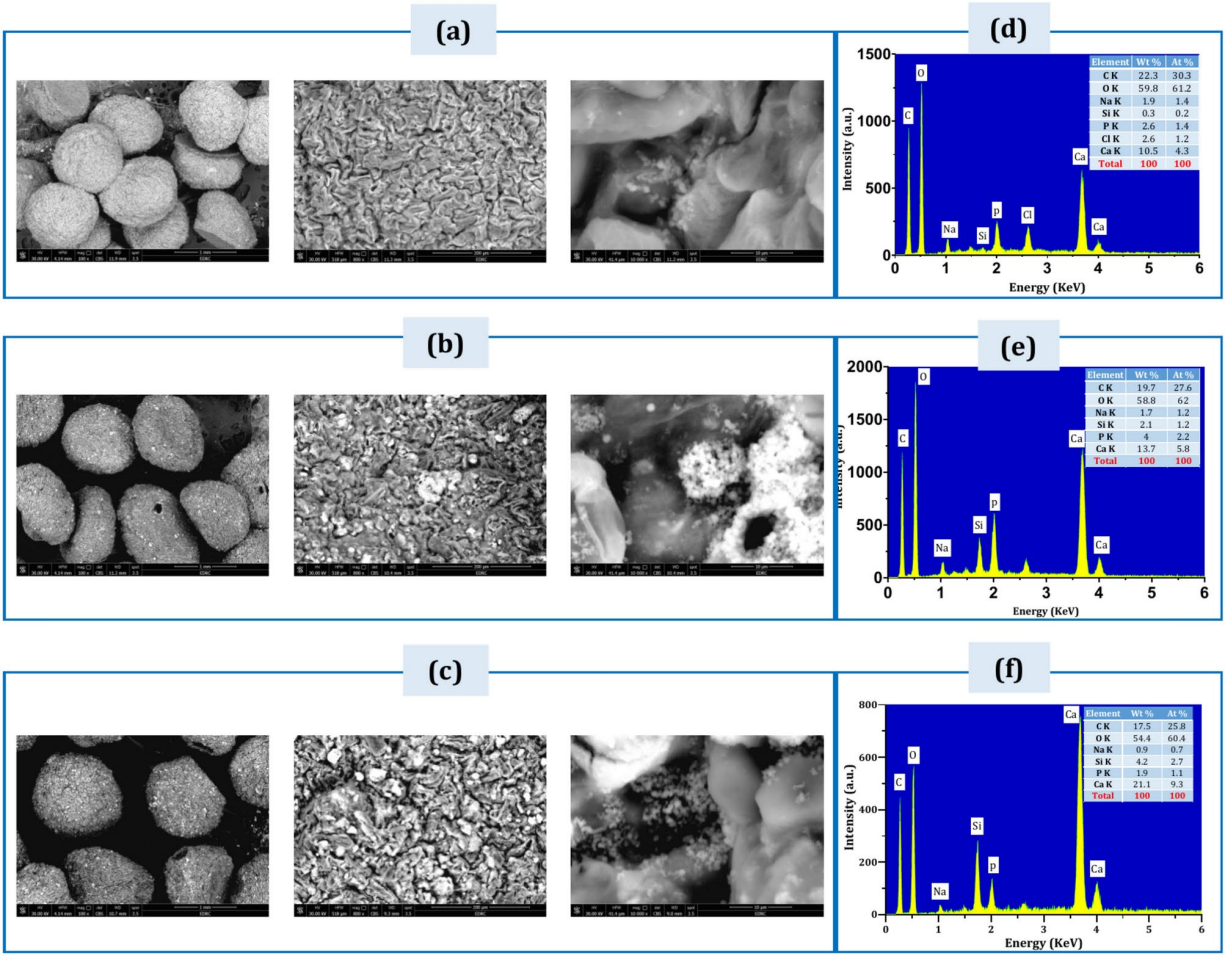
(SEM) images of, (**a**) B1, (**b**) S1, (**c**) S2, and EDXA profiles of (**d**) B1, (**e**) S1, (**f**) S2. Following 30 days of immersion in SBF.

Figure 7 showed SEM images of (a) B1, (b) S1, and (C) S2, and EDXA profiles of (d) B1, (e) S1, and (f) S2 after immersion in SBF for 30 days. All cations of carbon (C) and silica (Si) were decreased due to the degradation of polymers and BG in the SBF solution.

Calcium and phosphorous peaks were detected in the EDX analysis of all samples following immersion in (SBF). Calcium (Ca) and phosphors (P) were increased in each sample after the immersion process in SBF compared to their contents before immersion. Increases in calcium and phosphorus ratios indicate the deposition of HA covering the surfaces of the beads with a HA layer. The SEM findings indicated that the bead’s diameter is approximately 500 µm, smaller than the beads’ initial size before immersion in (SBF). The EDXA profile results showed that the peaks of silica decreased, and the peaks of calcium and phosphorus increased.

### Invitro study:

**Fig. 8:**
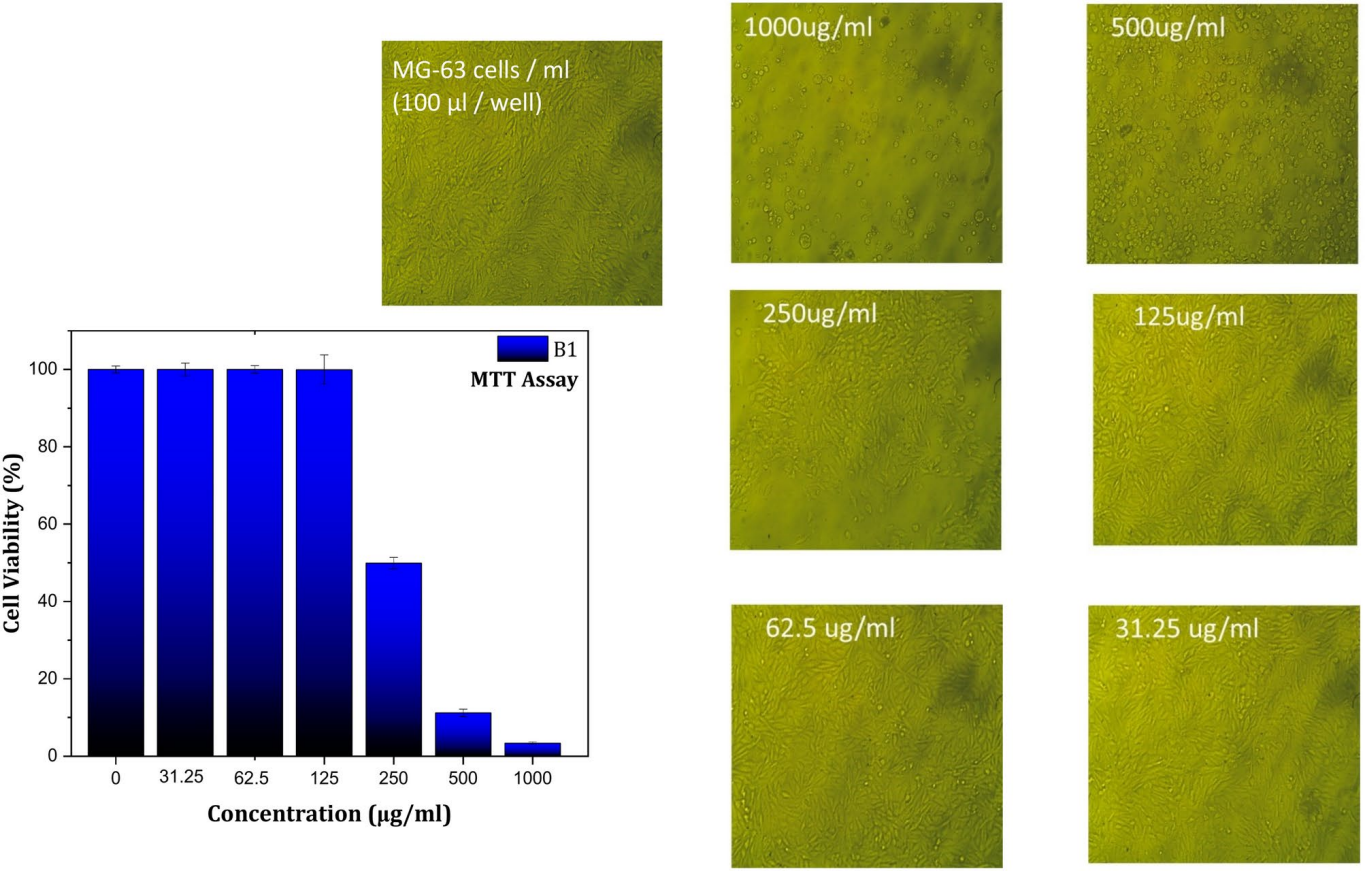
MG-63 cells viability results using MTT assay with different concentrations of B1 biocomposite beads.

**Fig. 9:**
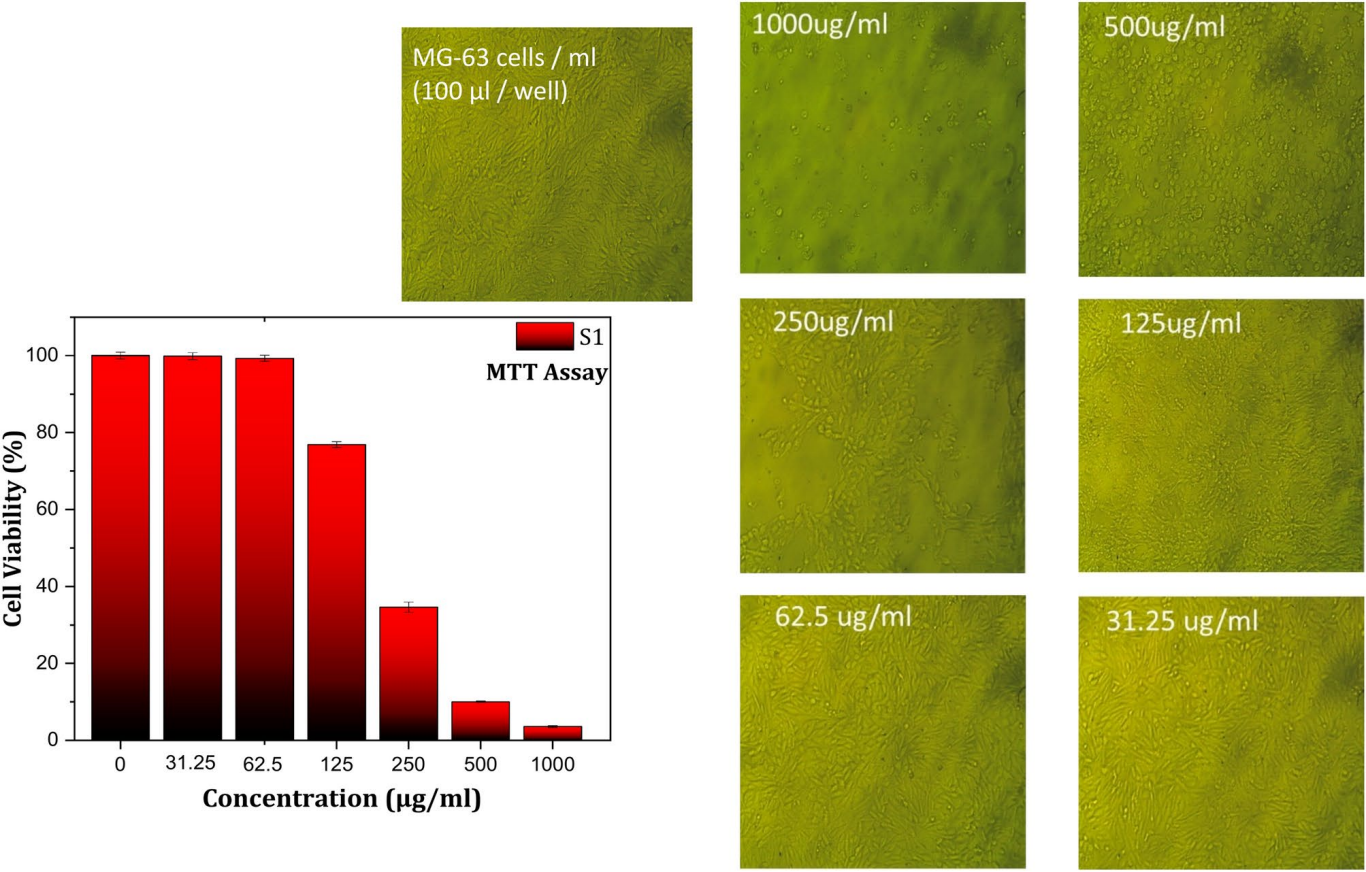
MG-63 cells viability results using MTT assay with different concentrations of S1 biocomposite beads.

**Fig. 10:**
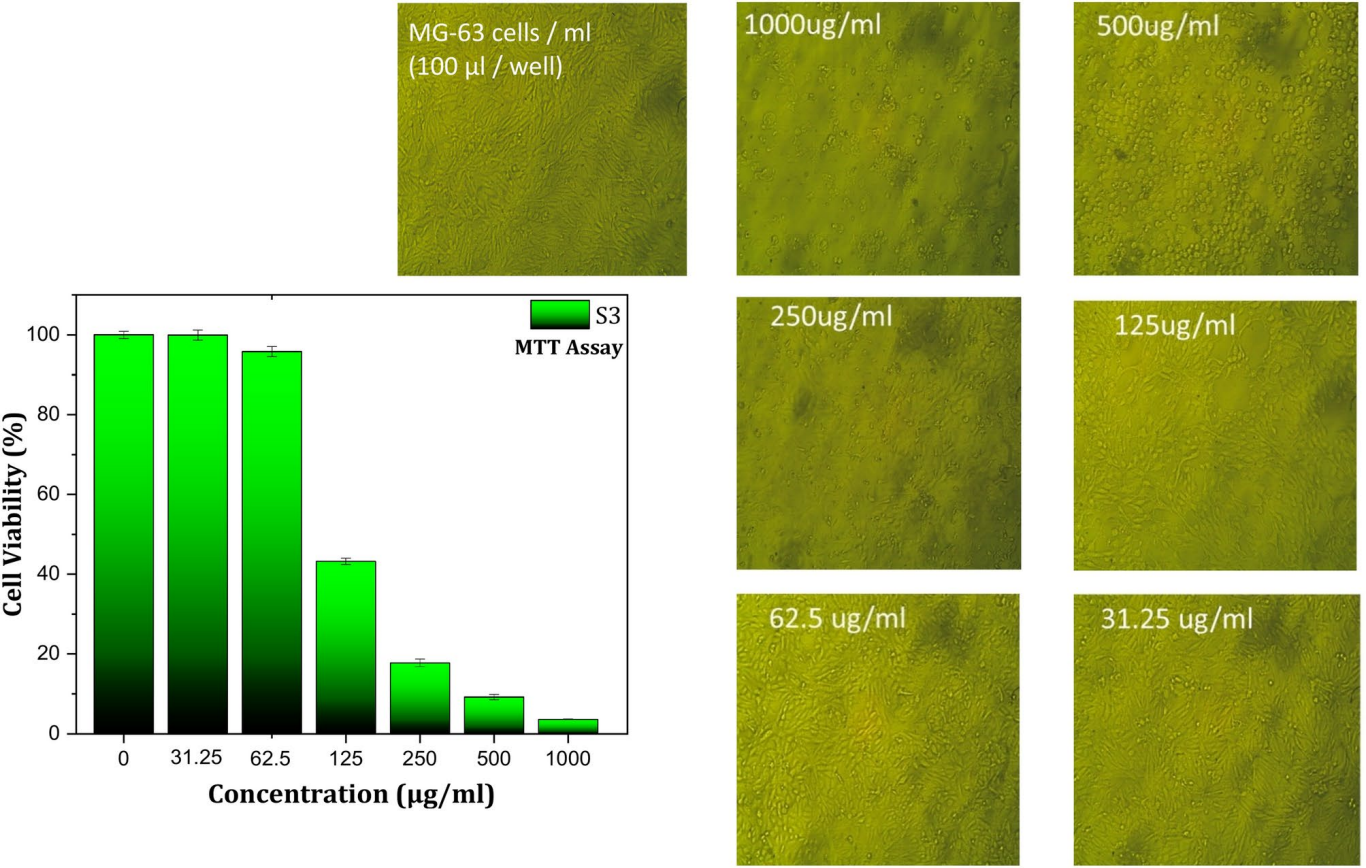
MG-63 cells viability results using MTT assay with different concentrations of S3 biocomposite beads.

Figures 8, 9, and 10 showed the results of the effect of different concentrations of B1, S1, and S3 biocomposite beads on the viability of 105 (100 µL/well) MG-63 cells using the MTT assay (Table 2). The results showed a significant decrease in cell viability as the concentration of each sample increased compared with the control. It’s noted that high cell death was detected on the MG-63 cells with S3 compared with B1 and S1 at the same concentration of each sample. Because of the significant methotrexate release from the complex structure of the biocomposite beads (S3) compared with B1 and S1, which agree with the drug release study results. Samples of B1, S1, and S3 were chosen for cytotoxicity studies to evaluate the relationship between BG content and methotrexate release. S3, with the highest BG content, demonstrated significant bioactivity, making it a key candidate for comparison.

**Table 2:** Drug release study of Methotrexate (MTX) from B1, S1, and S3 composite beads.

Samples ID	ug/ml	Toxicity %	Viability %	± SD	P- value	IC50 ± SD
**B1**	1000	96.60	3.39	0.29	7.40E-10	306.99 ± 2.72
	500	88.80	11.20	0.96	6.72E-09	
	250	50.08	49.91	1.47	4.78E-07	
	125	0.04	99.95	3.78	0.37	
	62.5	0	100	0.97	0.45	
	31.25	0	100	1.67	0.41	
**S1**	1000	96.43	3.57	0.28	2.08E-09	204.74 ± 4.55
	500	89.95	10.05	0.15	8.04E-10	
	250	65.39	34.61	1.32	4.41E-08	
	125	23.15	76.85	0.72	8.60E-10	
	62.5	0.70	99.29	0.81	0.03	
	31.25	0.13	99.87	0.91	0.33	
**S3**	1000	96.38	3.61	0.098	1.11E-09	116.16 ± 1.57
	500	90.78	9.21	0.67	5.92E-09	
	250	82.23	17.77	0.94	2.51E-08	
	125	56.74	43.25	0.81	2.69E-11	
	62.5	4.19	95.81	1.28	0.004	
	31.25	0.04	99.95	1.24	0.44	

The MTT assay revealed that the (IC-50 ± SD) of S3 is 116.16 ± 1.57 µg compared with 306.99 ± 2.72 µg and 204.74 ± 4.55 µg for B1 and S1, respectively. The reduction of cell viability of MG-63 cells may be related to the dissolution of drug (methotrexate) from B1, S1, and S3 biocomposite beads and its release through the MG-63 cell membranes, which aligns with earlier research^48^.

The composite beads produced incorporate various degradable polymers, such as polyvinyl, cellulose, and sodium alginate. The release of methotrexate from the MTX/BG-loaded PVA-CNC-SA biocomposite beads is influenced by the breakdown of these polymers. In the bead structure, the polymer ratio decreases progressively from B1 to S3, facilitating the degradation process and, in turn, the dissolution of methotrexate ^49^.

### Ultra-violet and visible spectroscopy (UV–VIS)

**Fig. 11:**
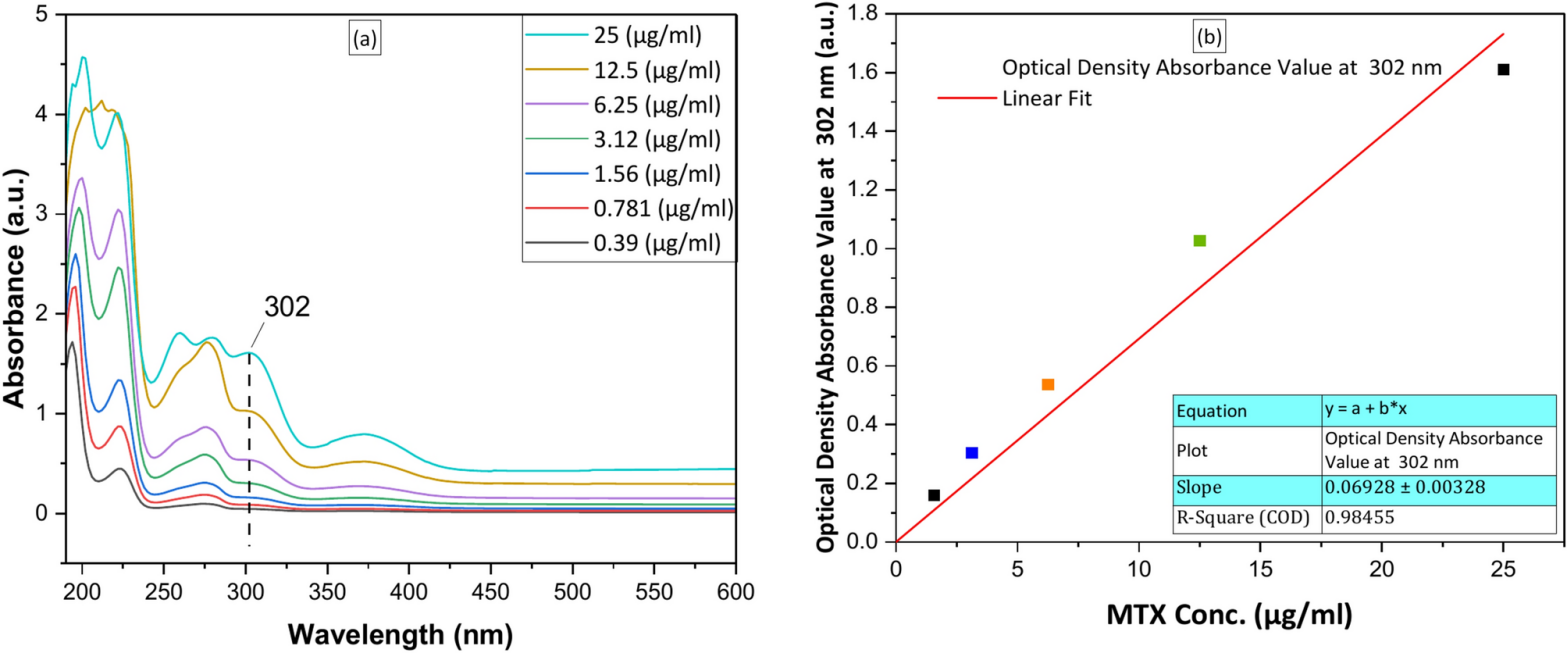
(**a**) UV–Visible absorption spectra of MTX concentration, (**b**) Calibration curve of the release drug MTX.

Figure 11a shows the UV–visible absorption spectra of the MTX solution. The absorption peak intensities of MTX were detected at a wavelength of 302 nm.

#### Calibration curve of the release drug methotrexate (MTX)

The UV–Vis absorption spectrometer (JASCO v-630) underwent calibration, with various standard drug concentrations (0.39 µg/ml, 0.78 µg/ml, 1.56 µg/ml, 3.12 µg/ml, 6.25 µg/ml, 12.5 µg/ml, and 25 µg/ml) in PBS solution. The highest wavelength (λ) absorption peak observed at 302 nm, corresponding to the MTX drug. The calibration curve (Fig. 11b) of the drug was adjusted to fit the straight line with the correlation coefficient (R^2^) = 0.98455.

The absorbance peak intensities of the samples MTX/BG-loaded PVA-CNC-SA biocomposite beads (B1, S1, S2, and S3) throughout the predetermined periods were detected by UV–visible absorption spectroscopy. The equivalent amount of drug was quantified using the corresponding calibration curves and listed in Table 3 as a percentage of MTX drug release.

**Table 3:** The Percentage of Methotrexate (MTX) drug released from samples.

Time (day)	B1		S0		S1		S2		S3	
	μg/ml	%	μg/ml	%	μg/ml	%	μg/ml	%	μg/ml	%
0	0	0	0	0	0	0	0	0	0	0
1	8.62	18.11 ± 0.17	8.53	16.69 ± 0.17	8.09	15.13 ± 0.16	5.138	9.54 ± 0.103	7.23	11.21 ± 0.14
2	5.63	29.94 ± 0.11	7.29	30.95 ± 0.15	6.42	27.14 ± 0.13	5.759	20.24 ± 0.12	7.85	23.39 ± 0.16
6	7.38	45.45 ± 0.15	7.20	45.05 ± 0.14	6.58	39.45 ± 0.13	7.953	35.01 ± 0.16	10.55	39.76 ± 0.21
16	8.31	62.92 ± 0.17	9.04	62.73 ± 0.18	10.43	58.96 ± 0.21	11.21	55.84 ± 0.22	12.80	59.61 ± 0.26
23	8.62	81.04 ± 0.17	9.37	81.07 ± 0.19	10.87	79.29 ± 0.22	11.73	77.64 ± 0.24	12.88	79.59 ± 0.26
30	9.02	100.00 ± 0.18	9.67	100 ± 0.19	11.07	100 ± 0.22	12.03	100 ± 0.24	13.15	100 ± 0.26
Total amount	47.58		51.105		53.489		53.843		64.479	
P value	.001		.003		.002		.001		.001	

*P-value < 0.05 was considered statistically significant. (Statistical software IBM—SPSS, version 27.0).

**Fig. 12:**
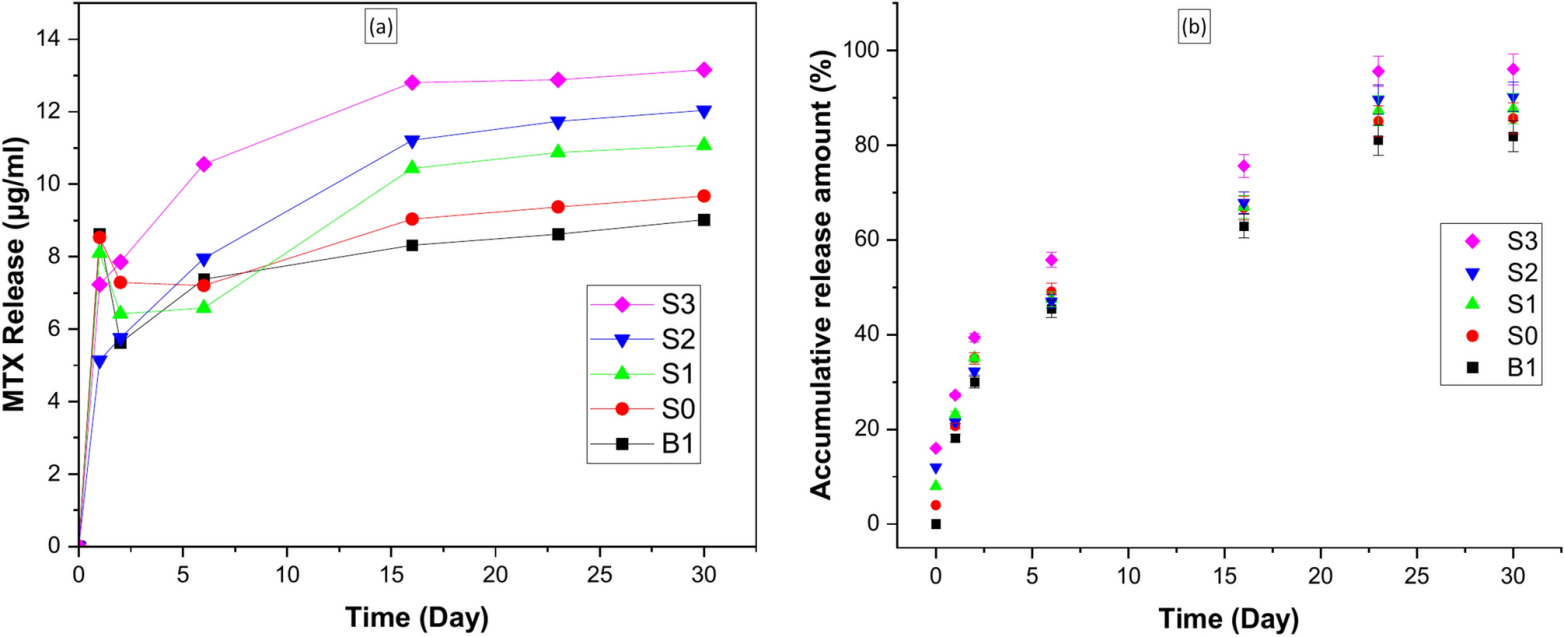
(**a**) the amount of release MTX concentration from samples at 30 days, (**b**) release profile of MTX in terms of the percentage (%) of MTX release as a function of time.

Figure 12a shows the simultaneous release of MTX from (B1, S0, S1, S2, and S3) and biocomposite beads over 30 days. Figure 12b describes the drug release profile (accumulative release) of MTX as a percentage (%) from B1, S0, S1, S2, and S3 biocomposite beads. The release profile for MTX initially showed a slow release, which steadily increased. The MTX release rate accelerated temporarily after 6 days and continued until 30 days.

The drug release profile was studied in three stages: an initial burst release (stage I), followed by accelerated release (stage II), and steady-state release (stage III). There was an initial burst release of MTX (at the end of two days: stage I) from B1, S0, S1, S2, and S3, with 29.94%, 30.95%, 27.14%, 20.24%, and 23.39% followed by a sustained release. At the end of 16 days (stage II), the release curves exhibited an increasing rate of MTX release (62.91%, 62.73%, 58.96%, 55.84%, and 59.61%), respectively. After 16 days (stage III), the amount of drug released from biocomposite beads B1, S0, S1, S2, and S3) remained constant or in a steady state (81.07%, 79.29%, 77.64%, and 79.59%), respectively. The profile is relatively similar for the four samples (S0, S1, S2, and S3), as would be expected. Since the MTX/BG-loaded PVA-CNC-SA biocomposite beads provide greater support for the drug than the MTX-loaded PVA-CNC-SA biocomposite beads (B1), due to the conjugation (carboxyl group) of the drug and the hydroxyl group of the BG and polymers^8^*.*

The drug release is governed by two factors: diffusion and degradation of the polymer^50^*.* MTX release by diffusion from S0, S1, S2, and S3 depends on the bioglass ratios in the biocomposite beads. The experimental results demonstrate that the release of MTX from S3, which contains a higher concentration of bioglass (0.6 g), is faster than from B1, S0, S1, and S2 biocomposite beads. This may be explained by bioglass’s high dissolution rate and MTX’s subsequent high release. Results confirmed that the release of the drug is influenced more by diffusion than by degradation. The in vitro drug release study was performed using MTX/BG-loaded PVA-CNC-SA as a carrier for MTX in the treatment of bone cancer.

Moreover, the kinetics of MTX release from B1, S0, S1, S2, and S3 biocomposite beads were figured out by matching against mathematical models as shown in Table 4. From the table, it is clear that both release kinetics from B1, S2, and S3 were matched with the Korsmeyer-Peppas model as they represented the highest (R^2^) values (0.95201, 0.99668, and 0.99615, respectively). This means that MTX was released from these fabricated beads through the hydration and degradation of polyvinyl and bioglass. The kinetic release from S0 and S1 were fitted to the Higuchi release model as its (R^2^) (0.9846 and 0.9807) value was the highest value, which means that the drug was released via quasi-Fickian diffusion (n < 0.45) (i.e. semi-controlled release).

**Table 4:** Release kinetics parameters of different MTX-loaded PVA-CNC-SA biocomposite beads.

Formula code	R^2^-value			Korsmeyer-Peppas model		n	k*
	Zero-order	Higuchi release model	Hixson-Crowell release model	R^2^-value	t_50_** (hours)		
B1	0.9409	0.9123	0.9306	0.95201	2156.403	0.06174	5.754
S0	0.9353	0.9846	0.9376	0.97765	2479.305	0.06348	6.123
S1	0.9582	0.9807	0.9548	0.95821	2189.021	0.15557	3.925
S2	0.9647	0.9741	0.9758	0.99668	1668.399	0.18407	4.063
S3	0.9781	0.9841	0.9620	0.99615	1499.305	0.26822	2.139

**n** is the diffusion exponent, k* is the release rate constant, **t**_50_** is the time required for 50% of the drug to be released, and R^**2**^-value is the value for the regression coefficient. (Statistical software IBM—SPSS, version 27.0).

## Conclusions

Herein, we developed a novel drug delivery system based on MTX/BG-loaded PVA-CNC-SA biocomposite beads for treating osteosarcoma. We prepared MTX/BG-loaded PVA-CNC-SA biocomposite beads using the dropwise method, varying the BG concentration. Bioactivity, drug release, and cytotoxicity were investigated. Results showed that the deposition of the HA layer on the surface of S2 and S3 confirmed their bioactivity. It was found that bioactivity is directly proportional to the BG amount. These results suggest the prepared samples are biologically reactive, demonstrating their potential to enhance apatite formation in bone tissue engineering. In-vitro drug release studies indicated the sustained release of MTX from the biocomposite beads for up to 30 days, which can consequently be used as a drug delivery system. High cell death was detected on the MG-63 cells with S3 compared with B1 and S1 due to the high release of methotrexate, which proves S3 biocomposite beads act as anticancer and can be used for the treatment of osteosarcoma with a reduction in the side effects of methotrexate. The results showed that the prepared biocomposite beads possess dual functions: they act as effective bioactive materials and serve as a promising carrier for methotrexate in osteosarcoma treatment. Additional research is needed to investigate the biocomposite beads in vivo applications in living organisms.

## Data Availability

The datasets generated during and/or analyzed during the current study are available from the corresponding author on reasonable request.

## Abbreviations

SA: Sodium alginate
CNC: Cellulose
PVA: Polyvinyl alcohol
SBF: Simulated body fluid
MTX: Methotrexate
BG: Bioglass
65S-BG: 65%SiO_2_-30%CaO- 5%P_2_O_5_
UV–VIS: Ultra-violet and visible spectroscopy
SEM: Scanning electron microscopy
XRD: X-ray diffraction
FTIR: Fourier transform infrared spectroscopy
EDXA: Energy-dispersive X-ray analysis
POECs: Polyelectrolyte complexes
